# Local Infrastructure Induces Edge Effect in Nocturnal Insects—A Case Study in the Donau-Auen National Park (Austria)

**DOI:** 10.3390/insects17070670

**Published:** 2026-06-26

**Authors:** Makrina Tsinoglou, Konrad Fiedler, Jacqueline Degen

**Affiliations:** 1 Institute for Biology and Environmental Sciences, Carl von Ossietzky University of Oldenburg, 26129 Oldenburg, Germany; 2 Chair of Behavioral Physiology and Sociobiology, University of Würzburg, 97074 Würzburg, Germany; 3 Department of Botany and Biodiversity Research, University of Vienna, 1030 Vienna, Austria

**Keywords:** nocturnal insects, moth community, protected area, light traps, edge effect

## Abstract

In protected areas, fragmentation and artificial light at night are usually present alongside changes in land type, from natural to agricultural or urban. We explored the intensity of edge effects on light trap responses of nocturnal insects at the margin of the floodplain forest in the Donau-Auen National Park in Central Europe, Austria. Specifically, we examined the abundance and biomass of nocturnal insects and characterized the community composition and diversity of moths with respect to the local habitat. In this study, 58 species were observed, with 21 unique records on the forest edge and nine in the interior. Traps in the forest interior harbored significantly higher nocturnal insect biomass. However, moth assemblages were more diverse at edge sites due to many singletons, attributed to individuals attracted from areas with open vegetation. Nine species (15.5% of total) were recorded later than expected given their summer flight periods, potentially reflecting the effects of ongoing climate change associated with warmer autumns. Overall, we observed higher moth species diversity at the forest edge, and insect biomass and moth abundance were higher within the forest. These findings underscore the urgent need to incorporate local anthropogenic landscape and climate change as synergically evolutionary drivers in future population and community-focused research.

## Simple Summary

Wild and domesticated pollinators are facing global declines caused by the synergy of numerous anthropogenic drivers. Protected areas are central to biodiversity conservation, but their edges are usually disturbed as natural habitats transition into agricultural or urban landscapes. Using light traps at the interior and edge of the floodplain forest in the Donau-Auen National Park, Austria, we examined the abundance and biomass of nocturnal insects as well as the community composition of moths. The forest interior had a significantly higher insect biomass, while the forest edge showed greater moth species diversity, likely due to individuals drawn from the surrounding open urban area. Notably, 15.5% of recorded species appeared unusually late in the season, suggesting that warming autumns driven by climate change are shifting moth flight periods. These results can have several long-term consequences on the local communities, ranging from altered populations to an expansion of specific species.

## 1. Introduction

Pollination assisted by animals is one of the most important and irreplaceable ecosystem services [1]. It is estimated that 88% of all angiosperm species and 35% of global crops are pollinated by animals [2,3]. Moths (Lepidoptera) are one of the most important nocturnal pollinators of a wide range of plant species [4,5,6,7,8], particularly the two most numerous moth families, Noctuidae (owlet moths) and Geometridae (geometrid moths).

In recent decades, there has been a decline in wild as well as domesticated global pollinators caused by the synergy of many factors such as land use changes, fragmentation, urbanization, use of agrochemicals, pathogens, and climate change [9,10]. Although a wide range of sampling methods are used to assess arthropod biodiversity and abundance (e.g., Malaise traps, light traps, pitfall traps), the evidence consistently points to declines in terrestrial insect abundance, biomass, and species richness across taxa and regions [11,12,13,14]. A recent study reports a 22% decline in total butterfly abundance across the United States over just 20 years [15]. Moths in particular have shown severe long-term declines in Britain, with 21% of studied macro-moths declining by more than 30% per decade [16]. While arthropod biomass and species richness have been shown to decline in both grasslands and forests, largely attributed to landscape-level drivers [13], more recent evidence suggests that moth declines are most pronounced in broadleaf woodland habitats [17]. Although the precise causes of insect decline are not well documented geographically and taxonomically, the reported population declines of abundant species (from major orders such as Coleoptera, Hymenoptera, and Lepidoptera) indicate the need for urgent monitoring efforts [18].

The presence of urban infrastructure is usually coupled with artificial light sources that introduce an additional stressor for nocturnal pollinators, viz. light pollution, which has changed night skies across the globe [5,19,20,21]. Artificial night light can disrupt a number of biological activities (e.g., foraging, reproduction, migration) by disturbing the natural diurnal, seasonal and lunar cycles of light that a wide range of organisms use as timing cues [22,23,24]. Light pollution is still globally increasing [25,26,27] and has been proven to be a driver of environmental change [22,28]. Its impact spans from the cell level [29] to individuals [23] and communities [30]. Moths are most famous for being attracted to artificial light sources [31,32]. However, there are alarmingly many more direct and indirect effects recorded in adult moths, such as inhibition of feeding, higher predation and increased tortuosity of flight [33,34,35]. Effects are also present across all stages in moth life cycles (e.g., larva: reduced body mass) and even communities are affected through many different mechanisms [5]. Alteration in the perception of photoperiod, manifested as changes in the timing or prolongation of activities, can cause shifts in interspecific interactions and pollination services, while ongoing climate change represents an additional threat by driving phenological mismatches and shifts in species distributions through rising temperatures and increasing extreme weather events [5,23,28,36,37,38].

Protected areas are among the main tools for safeguarding biodiversity from human-induced threats and are geographically defined areas that are legally designated and managed for nature conservation [39,40]. Protection management strategies usually focus on the conservation of plants and vertebrates, while insects are often neglected, although they represent more than 80% of all animal species [41,42,43]. Studies showed that insects even face a great number of threats inside protected areas with natural system modifications recorded as major threats for Lepidoptera [41]. Additionally, protected areas can experience disturbance by artificial light originating from authorized activities within their boundaries, from sources near the area’s edges or from skyglow caused by nearby densely urbanized regions [44]. Insect sensitivity to light pollution and habitat loss due to agricultural and urban changes calls for focused research on monitoring insect communities inside both protected areas and their buffer zones [41,45].

The aim of this study was to explore the intensity of such edge effects on light trap responses of nocturnal insects at the margin of the floodplain forest in the Donau-Auen National Park in Central Europe, Austria. Based on light trap catches, we examined the abundance and biomass of nocturnal insects and determined the community composition and diversity of moths with respect to the local habitat. Using this data, we were able to investigate how varying surrounding habitats influence nocturnal insect captures in light traps, assess differences in moth community composition at a fine spatial scale, and evaluate whether the forest edge exhibits reduced abundance and diversity. As the fieldwork was conducted in autumn, we were able to also examine the effects of phenological progression during this late season on moth assemblages.

## 2. Materials and Methods

### 2.1. Study Area and Field Sites

**Figure 1:**
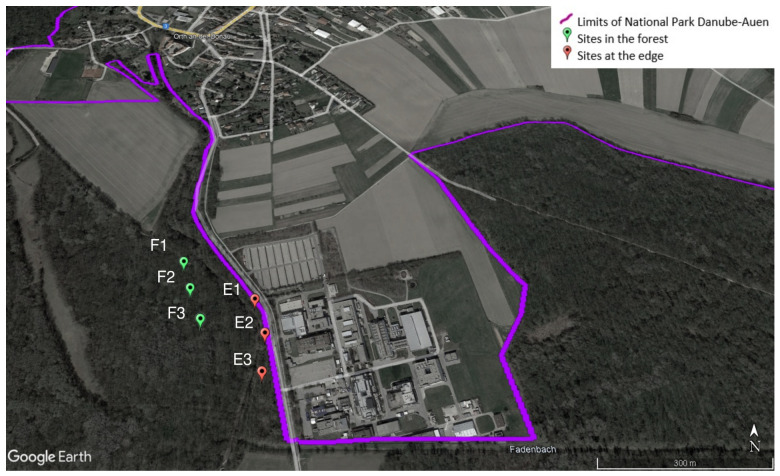
Map of the sampling area and light-trapping sites at the Danube-Auen National Park near Orth at the Donau, in Austria.

The study area is part of the Danube-Auen National Park, near the village of Orth an der Donau (Lower Austria, Austria) (48.1451° N, 16.7026° E) (Figure 1). The study area is characterized by a deciduous lowland floodplain forest. While this part of the forest no longer experiences near-annual flooding by the Danube River, due to a dam constructed in the 1870s, it remains strongly influenced by periodically high groundwater levels. The tree layer vegetation consists mainly of species such as oak (*Quercus robur*), maple (*Acer campestre, platanoides* and *pseudoplatanus*), ash (*Fraxinus excelsior*) and poplar (*Populus alba*). The shrub layer comprises privet (*Ligustrum vulgare*), hawthorn (*Crataegus* spp.), bird cherry (*Prunus padus*), common dogwood (*Cornus sanguinea*) and many others. The Donau-Auen National Park is an ideal location, as it features distinct natural and managed zones. While the outer zones experience the most disturbance as they are used by the public for recreational purposes, inner zones remain comparatively undisturbed. The drastic habitat change that is present at the limits of the National Park from unmanaged forest to settlement structures likely causes severe edge effects, as many studies have shown that forest edges affect a great number of forest species and ecological processes [46]. Two groups of sites were selected depending on their distance from the borders of the National Park and in both groups the vegetation composition was the same regarding both closed canopy and shrub species.

**Figure 2:**
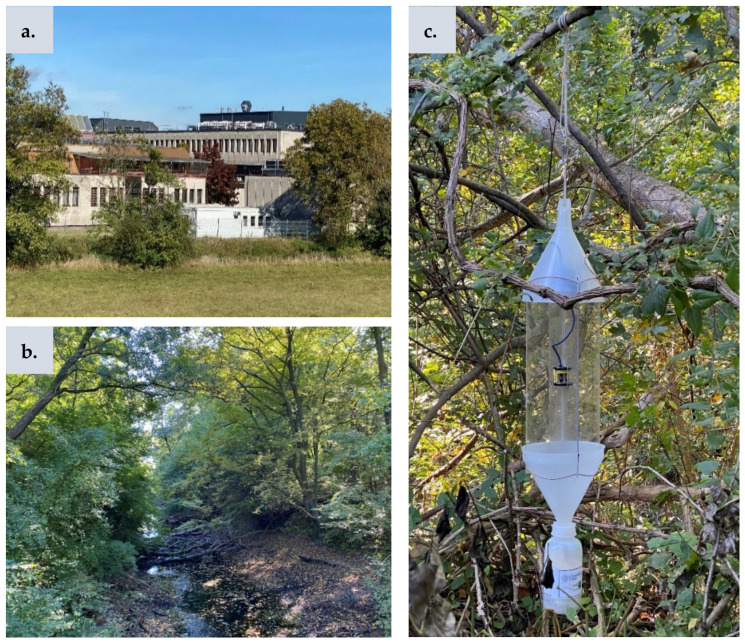
(**a**) Factory adjacent to the edge of the National Park; (**b**) tree canopy and low vegetation representative of the study area; (**c**) example of a light trap used in this study.

Three sites were selected directly at the edge of the National Park, less than 10 m away from a street illuminated by streetlights and an adjacent pharmaceutical plant (Figure 1 and Figure 2a). In the immediate vicinity of the sampling sites, streetlights were switched off around 10 pm, and most were sodium vapor luminaires emitting yellowish light. However, the industrial buildings were illuminated throughout the night by a mixture of light sources varying in type, intensity, and spectral composition (Figure 2a). Three forest sites were selected under closed woodland canopy (>20 m tall), away from artificial lights and with low levels of human disturbance, while belonging to the same forest type (Figure 1). Night-time sky brightness differed between the forest zones, with relatively dark sky in the forest interior (VIIRS 2021: 4.2 nW/cm^2^·sr) and higher values at the forest edge (VIIRS 2021: 8.19 nW/cm^2^·sr) (data from http://www.lightpollutionmap.info (accessed on 9 December 2025), [25]. All sites were situated at least 100 m apart from each other. We conducted 7 nightly sampling rounds during a period of 7 weeks from early October to mid-November 2021. The sampling nights were: 4 and 5 October (Round 1), 11 and 12 October (Round 2), 13 and 14 October (Round 3), 19 and 20 October (Round 4), 3 and 4 November (Round 5), 9 and 10 November (Round 6), and 15 and 16 November (Round 7).

### 2.2. Light Trapping and Identification

Automated light traps were placed about 1.5 m above ground (Figure 2c). Each trap was equipped with one multi-spectral LED lamp [47], powered by a 12 V battery pack. This LED lamp emits irradiation peaks at 368 nm (UV), 450 nm (blue), and 520 nm (green), corresponding closely to the peak sensitivity of moth eye receptors. The lamp emitted 54% of its total irradiance (1.43 W m^−2^ at 50 cm) in the UV range (300–400 nm) and 45% in the blue and visible light (401–650 nm). The basic design of the traps (Figure 2c) followed Axmacher & Fiedler, 2004 [48]. These light sources have earlier been shown to attract moths from a rather small radius [49]. Sampling was only conducted in favorable conditions (mild nights without strong wind or heavy rainfall) and scheduled to avoid full moon periods, as all these factors can have a major influence on moth catch sizes [50]. Sampling started at dusk (around 17:00–18:00) and ended at midnight. Moth flight activity usually gets reduced during the late course of the night as air temperature drops substantially in temperate ecosystems, especially during the cold months of October and November. The average air temperature on the sampling days ranged from 6 to 20 °C (taken from a nearby weather station: https://www.wunderground.com/dashboard/pws/IORTHA1 (accessed on 25 November 2021)). Each night the light traps were used, the sampling containers were equipped with liquid chloroform, which evaporated slowly. Insects attracted to the light trap were captured by falling into the sampling container, where they were anesthetized by the chloroform. The following morning, they were transferred to a specially designed jar containing hydrogen cyanide held securely at the bottom in a cork compartment. Subsequently, fresh mass was recorded for all insects combined and for moths specifically. All moths were then pinned and identified according to species level. Afterwards they were labeled and preserved in a freezer at −20 °C. Since a few moth specimens were in poor condition, we used DNA barcoding techniques for their identification. DNA extraction was performed using legs of the moths. Generating the COI barcode sequences followed standard laboratory protocols using innuPREP DNA Micro Kit (Analytik Jena, Jena, Germany) and BigDye^®^ Terminator v3.1. Sequencing was conducted on a 3130 × l Genetic Analyzer (Applied Biosystems, Waltham, MA, USA). Obtained sequences were uploaded on the database BOLD (boldsystems.org) along with data on sampling location, date and images of the specimens. For identification, they were matched with reference sequences through a BLAST version 2.12.0 search [51].

### 2.3. Statistical Data Analysis

Statistical analyses were conducted using R version 4.1.1 (R Core Team, 2021) [52]. Prior to the main analysis, we tested whether the three sites within each habitat type (forest and edge) could be treated as replicates by comparing biomass and abundance among sites using Kruskal–Wallis tests [53]. Moth biomass, non-moth insect biomass and total insect biomass did not differ significantly among the three forest sites (Kruskal–Wallis: moth biomass: H = 0.92, *p* = 0.63; non-moth biomass: H = 2.32, *p* = 0.31; all insect biomass: H = 0.124, *p* = 0.94) or among the three edge sites (Kruskal–Wallis: moth biomass: H = 1.165, *p* = 0.56; non-moth biomass: H = 1.299, *p* = 0.52; all insect biomass: H = 1.61, *p* = 0.45). Moth abundance also did not differ significantly among forest sites (H = 0.18, *p* = 0.91) or among edge sites (H = 2.492, *p* = 0.29), justifying the pooling of data by habitat type for all subsequent analyses. We then examined differences between habitat types (forest vs. edge) and sampling rounds with respect to total insect biomass, moth biomass, and moth abundance using ANOVA. Dependent variables were log-transformed prior to analysis to meet normality requirements.

Diversity indexes (specifically, species richness and exponential of Shannon index) were calculated for the accumulated moth catches at each site using the package ‘vegan’ [54]. Differences in community composition per sampling night were assessed through non-metric multidimensional scaling (NMDS) based on Bray–Curtis dissimilarities (after square-root transformation of moth counts) tested for significance by a PERMANOVA using the adonis function within ‘vegan’ version 2.6-10 [54]. A test for homogeneity of multivariate dispersions using the betadisper command within ‘vegan’ for the two factors of site position and season was applied [54]. Since some nightly samples were rather sparsely populated, we added a dummy species with pseudo-abundance of 1 to all of them [55].

Το quantify and compare gamma diversity across the two groups of sites we used the ‘iNEXT’ version 3.0.1 (iNterpolation/EXTrapolation) algorithm, an R package that provides functions to compute and plot sample size and coverage sampling curves, along with confidence bands [56,57]. Raw abundance for each moth species aggregated in the two groups of sites (forest vs. edge). We used the sample-size-based R/E curve (type = 1) to examine whether sampling effort was sufficient and how diversity is expected to increase with additional sampling. To estimate potentially undetected species richness in each habitat type, we calculated the bias-corrected Chao1 estimator (Chao1-bc) for the forest and edge habitats, both overall and separately for the early and late seasons [58]. The Chao1-bc estimator was chosen over the standard Chao1 following an assessment of singleton proportions, as it is recommended when the percentage of singleton species exceeds 33% [58]. The proportion of singletons exceeded this threshold at the forest edge overall (46.9%) and in the early season (48.9%), therefore Chao1-bc was applied consistently across all groups to ensure comparability.

Finally, to further explore differences in species composition between the two groups of sites we used the package ‘VennDiagram’ version 1.7.3 [59]. Aiming to determine which species can serve as indicators regarding the ecological group and season we performed an Indicator Species Analysis [60] using the function indval() in ‘labdsv’ version 2.1-0 [61]. Here, the factor ‘Season’ was added with two levels: ‘early’ = until 20 October (i.e., up to the 4th sampling round) and ‘late’ = after 20 October (viz. the 5th to 7th sampling round). All data were again square-root transformed. The R script and all raw data used in this study are available in the Supplementary Materials.

## 3. Results

We captured a total of 875 moths belonging to 58 species (10 families) in the study area (Appendix A). The most dominant species, mainly at the sites in the forest interior, (in descending order) were: *Ptilophora plumigera*, *Epirrita dilutata*, *Poecilocampa populi*, *Operophtera brumata*, *Agrochola macilenta*, *Colotois pennaria* and *Erannis defoliaria*. All these moths represent typical late-autumn species of deciduous forests in Central Europe.

Twenty-five species (43.1% of the total) were observed in just one individual over the entire period (‘singletons’). Nine species (15.5% of the total) represented unusually late records of moths that have their main flight period in summer (*Abrostola triplasia*, *Camptogramma bilineata*, *Cosmia pyralina*, *Eupithecia inturbata*, *Hoplodrina octogenaria*, *Hypsopygia costalis*, *Pandemis dumetana*, *Pleuroptya ruralis*, and *Yponomeuta plumbella*).

**Figure 3:**
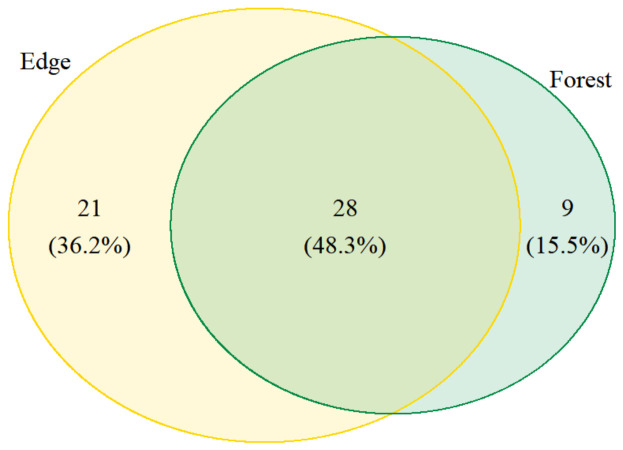
Venn diagram depicting the numbers of species unique to edge sites, forest interior sites, and species shared between both groups of light trap sites.

As expected, species richness changed over the sampling period. As the temperature dropped with the season’s progress, the late-summer and early-autumn species disappeared with time and the true autumn and winter moths emerged (Table 1). Overall, we recorded 50 species in the early season (‘early’ = from 4 until 20 October) and 16 species in the late season (‘late’ = 20 October to 16 November). Species richness was higher at the forest edge with 49 moth species while inside the forest we captured only 37 species (Table 1, Figure 3). Less than 50% of the total species (28) were common to both the forest interior and edge. We observed more species uniquely at the edge of the protected area (36.2% of the total), while only nine species were unique to the forest (Figure 3). Most of these unique records in both groups of sites were singletons, or species represented by only one individual (seven inside the forest, 18 on the edge).

**Figure 4:**
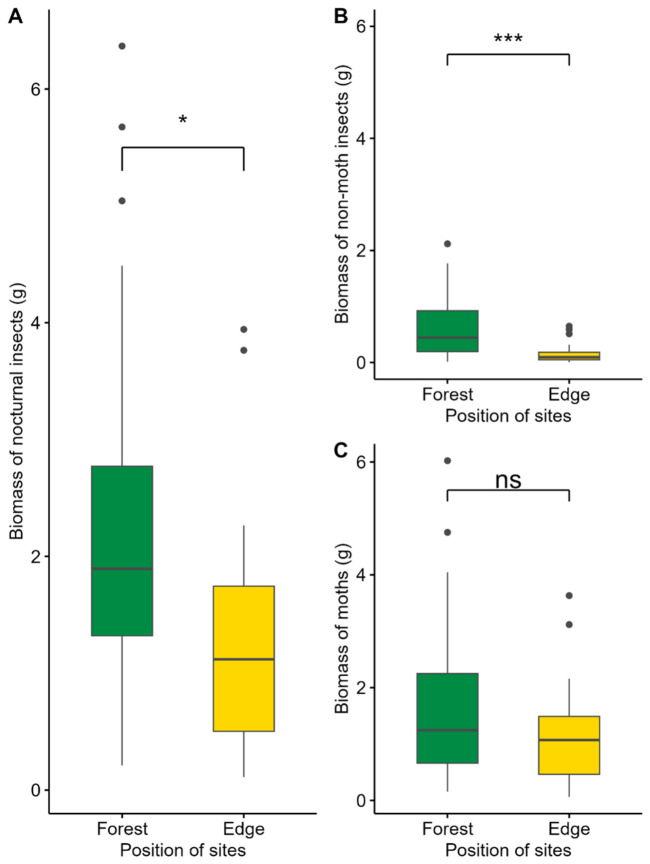
Biomass of captured insects per sampling round and site: (**A**) total insect biomass, (**B**) biomass of non-moth insects, and (**C**) biomass of moths. Boxes represent the interquartile range with the median shown as a black line; whiskers extend to the minimum and maximum values within 1.5 times the interquartile range, and circles denote outliers. Asterisks indicate statistically significant differences (* *p* < 0.05, *** *p* < 0.001, ns = not significant).

The position of traps, at the forest edge or interior, had a significant effect on overall biomass and abundance of insects (Figure 4). The biomass of total nocturnal insects (moths and other nocturnal insects combined) was significantly higher in the forest interior than at the edge (ANOVA: F1,40 = 4.755, *p* = 0.035) (Figure 4A).

**Figure 5:**
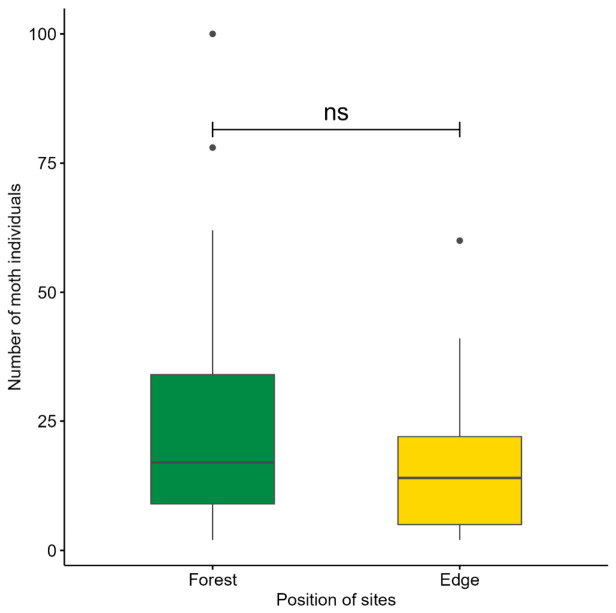
Number of individual moths per light trap night and site by light trap position. Boxes represent the interquartile range with the median shown as a black line; whiskers extend to the minimum and maximum values within 1.5 times the interquartile range, and circles denote outliers. Significance level: ns = not significant (*p* > 0.05).

Inside the forest non-moth insect biomass per trap night was significantly higher than at the edge (ANOVA; F1,40= 13.18, *p* < 0.001) (Figure 4B). On the contrary, neither moth abundance nor biomass showed any significant difference between forest interior and forest edge (abundance; F1,40 = 1.686, *p* = 0.202; biomass: F1,40 = 1.497, *p* = 0.228, abundance and biomass of moths) (Figure 4C and Figure 5).

Observed moth species richness per site, aggregated over all available nightly samples, varied from 21 to 34 species, with no obvious disparity between forest edge and interior sites (Table 2). Effective number of moth species (i.e., exponential Shannon’s H’) ranged from 7.96 to 14.43 and was only weakly correlated to observed richness (r = 0.421).

**Figure 6:**
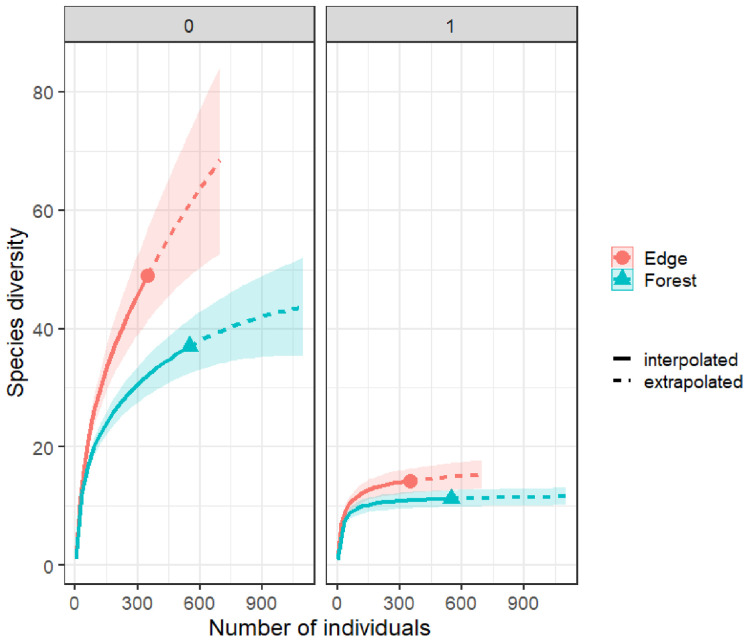
Sample-size-based rarefaction and extrapolation (R/E) curves (type = 1). These curves plot moth species diversity at forest edge and forest interior trap positions, pooled across three sites. Solid lines represent rarefaction (interpolation) based on observed data, and dashed lines represent extrapolation up to double the reference sample size. Shaded areas denote 95% confidence intervals. Left panel: q = 0, i.e., species richness: right panel: q= 1, i.e., exponential Shannon entropy H’.

When aggregated over all three sites per trap position (forest edge vs. interior), moth species diversity was markedly higher at forest edges than in forest interiors (Figure 6). This difference was most pronounced for observed species richness (Hill number q = 0), where sample-size-based accumulation curves did not reach an asymptote, indicating incomplete sampling.

Accumulation curves for higher Hill numbers (q > 0) approached asymptotes, suggesting that estimates of diversity weighted toward common species were more stable and that observed species richness was strongly influenced by singleton individuals (Figure 6). Estimated total species richness was considerably higher at the edge (Chao1-bc = 99.6) compared to the forest interior (Chao1-bc = 44.9). Sampling completeness was lower at the edge (49/99.6 = 49%) than in the forest interior (37/44.9 = 82%), indicating that the edge community contained a greater proportion of undetected species despite the higher observed richness. When analyzed by season and site group, the pattern was driven primarily by the early season, where estimated richness was markedly higher at the edge (Chao1-bc = 80.5, completeness = 52.2%) than in the forest interior (Chao1-bc = 35.4, completeness = 81.9%). In the late season, both habitats showed high sampling completeness (edge: 88%, Chao1-bc = 12.5; forest: 96.6%, Chao1-bc = 14.5) and low singleton proportions (27.3% and 14.3% respectively), suggesting a more homogeneous and thoroughly sampled community. These results indicate that the greater undetected diversity at the forest edge is primarily driven by a large pool of rare and transient species in the early season (Table 3).

**Figure 7:**
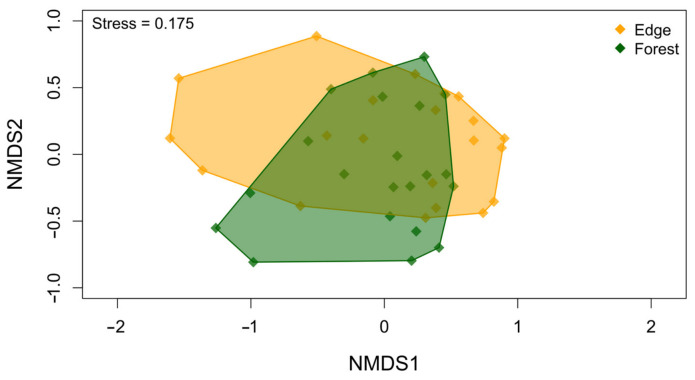
Non-metric multidimensional scaling (NMDS) ordination plot of nightly moth samples colored by light trap position based on Bray–Curtis dissimilarity, with convex hulls. Stress value of the two-dimensional solution is 0.175.

**Figure 8:**
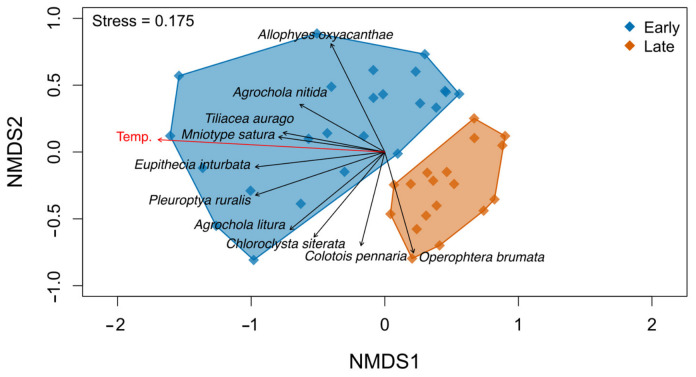
Non-metric multidimensional scaling (NMDS) ordination plot of nightly moth samples, based on Bray–Curtis dissimilarity. Colored by season (early, late) with convex hulls, with species abundances superimposed as vectors (only for the 10 moth species with significant contribution to the ordination pattern, *p* < 0.001); significant environmental variables overlaid as vectors (*p* < 0.05) (temp. = mean air temperature of the sampling date) and as categorical factors (early and late season). Stress value of the two-dimensional solution is 0.175.

Interestingly, when the effects of light trap position and season on species composition were evaluated simultaneously using a two-way PERMANOVA, both factors showed statistically significant main effects (position: F1;38 = 2.51, *p* = 0.02; season: F1;38 = 15.82, *p* = 0.001). In contrast, the interaction between season and position was not significant (F1;38 = 0.898, *p* = 0.48), indicating that the effect of trap position on species composition was consistent across seasons. As indicated by a strong overlap of data clouds in the ordination diagram (Figure 7), moth species composition varied only marginally between forest edge and interior sites. Forest edge samples showed a slightly broader dispersion than those from the forest interior. However, seasonal progress was a highly significant factor to explain variation in moth community composition (Figure 8).

However, apart from a shift in typical species composition (position of the data cloud in the NMDS ordination, also corroborated by the PERMANOVA), we observed a substantial decrease in dispersion among samples from early to late autumn (Figure 8). A test for homogeneity of multivariate dispersions revealed that dispersion was homogenous when samples were grouped by position of sites (F1;40 = 0.89, *p* = 0.35) but dispersion was inhomogeneous when grouped by season (F1;40 = 13.6, *p* < 0.001).

The analysis of indicator species revealed that only three species (*Agrochola macilenta*, *Colotois pennaria*, *Asteroscopus sphinx*) mainly occurred inside the forest, while no species were characterized as indicators for the edge sites (Table 4). A substantial set of species was characteristic for the late-season aspect (after 20 October), while others were mostly present in the early season (from 4 until 20 October). *Ptilophora plumigera*, *Poecilocampa populi* and *Operophtera brumata* are well-known characteristic winter moth species that were emerging massively only when the weather conditions became mild again after the first frost.

## 4. Discussion

The profile of the observed moth community was very typical for a deciduous forest in Central Europe during autumn (Appendix A). The dominant species were late autumn species present mainly in the interior of the forest. On the whole, 58 species were observed during the study with 21 unique records on the forest edge and nine unique records on the interior (Figure 3). Our results show that the position of the light traps had a clear effect on abundance and richness of nocturnal insects. Specifically, the forest interior, where the traps were under dark sky and tall tree canopy harbored a significantly higher biomass of nocturnal insects (Figure 4). This effect could mainly be attributed to non-moth insects, as when tested separately non-moth insects had significantly higher biomass in the forest interior, while moth abundance and biomass showed no such difference (Figure 4 and Figure 5). On the contrary, moth assemblages tended to be more diverse at the edge sites. This pattern was more pronounced regarding observed species richness than with exponential Shannon diversity. We attribute this to the high number of singletons, especially at the edge sites. The disparity of results between moths and non-moth insects further suggests that moths are on average more mobile than other nocturnal insects, so that adverse edge effects on insect biomass were less obvious among moths.

Overall, 25 species (43.1% of the total) were observed in just one individual over the entire period (‘singletons’). Of the 25 singleton moth species, 18 were recorded at the forest edge, while only seven were recorded in the interior. In a recent study focusing on the effect of light pollution on forest edges regarding bat responses, the authors observed lower numbers of individual moths on the light-exposed edges but higher biomass, indicating that larger Lepidoptera were more strongly attracted by light [62]. One of the main predators of moths are bats and they have been demonstrated to be affected by artificial light at night, yet with some bat species being more tolerant of light disturbance [62,63,64]. An interesting study also showed that LED streetlights reduce moths’ behavior against bat calls [63]. Changes in behavior of predators and prey (bats and moths) may ultimately cause shifts in the balance in the ecosystem.

Ries et al. (2004) [46] described four fundamental edge effect mechanisms that arise from altered resource distributions and species interactions, leading to distinct ecological patterns. A positive effect response is predicted when the habitat borders a lower-quality habitat, as is present in our study area. The insect edge-biased distribution is widespread across agricultural systems and different scales, with many environmental factors (e.g., wind patterns, microclimate, and vegetation) that are potentially responsible for this phenomenon [65]. As the response and the extent of the edge effect on populations and species richness is closely related to individual movement behavior and the distance an animal group can cover, it is important to consider that insect responses can vary greatly depending on life traits. Even in our small study, we observed higher moth species diversity on the forest edge, while overall insect biomass and abundance of moths were lower there. This can be attributed to individuals being attracted from a larger distance, directly at the edge of the forest, from different habitats. These moths could possibly fly from the nearby suburban area or agricultural fields towards the artificial illuminated area and the adjacent forest niche. Additionally, light from the traps can possibly reach further on the edge sites than those under the dense forest canopy and undergrowth vegetation present in the forest interior [49].

Another factor that could have influenced the abundance and richness of moths is the presence of streetlights and industrial buildings at the edge of the protected area, as these structures are sources of artificial light at night. Although the sites were positioned fairly locally, the values retrieved from the light pollution map revealed that sky brightness was substantially higher at the edge of the forest compared to the interior (VIIRS 2021: forest: 4.2 nW/cm^2^·sr, edge: 8.19 nW/cm^2^·sr) [25]. Street lighting can strongly affect local insect populations, as has been shown before; for example, street lighting has been reported to reduce the abundance of moth caterpillars compared to unlit areas [66]. It is even more alarming considering that light pollution has been present in local habitats and ecosystems for many decades and seems to be even acting as an evolutionary driver [67]. Species can adapt to the new conditions in urban areas, with reduced flight-to-light behavior of urban populations compared to rural ones being a striking example demonstrated by Altermatt & Ebert (2016) [68]. However, previous field-based studies have provided mixed results regarding species richness and abundance, showing not one universal direction of responses to light pollution [66].

In our study, species richness and composition followed a temporal phenological pattern as well. During the first sampling round (two sampling nights) in the early autumn season the number of species recorded in the light traps was the highest, at 41 species. Progressing though the season, the number of species per sampling round fell to 14 species on average. This is what was expected, as when temperatures are dropping, the phenological profile of the moth community changes. The last few late-summer and early-autumn species were then replaced by the true winter species. In our catches, *Ptilophora plumigera*, *Poecilocampa populi*, *Operophtera brumata*, *Asteroscopus sphinx* and *Erannis defoliaria* were the dominant winter species. These are characteristic winter moth species that emerged massively when weather conditions became mild again after the first frost. So, the high abundance of these species and the gradual disappearance of early-season species explain the high indicator values for the late season (Table 4).

Only three species emerged as indicator species for the forest interior (*Agrochola macilenta*, *Colotois pennaria* and *Asteroscopus sphinx*), while our analysis revealed no indicator species for the forest edge (Table 4). This indicates that there were no specific species characteristic of the forest edge ecotone. Notably, although the majority of species were specialists and generalists found in deciduous forests and wetlands, there was a small number of species that are open habitat specialists or synanthropic (*Ammoconia caecimacula*, *Eupithecia ericeata*, *Cadra furcatella*, and *Hypsopygia costalis*). These species were caught in one individual and mainly on the forest edge, suggesting that they were attracted from nearby open space habitats and gardens (Appendix A). On the other hand, it seems that the three ‘indicator’ species are not dispersive and avoided entering the more disturbed edge habitats. Nevertheless, we should keep in mind that some observations could be limited to the specific area because of the small sampling scale of our study. It is relevant also to point out that Luque et al. (2007) [69] reported that interannual variation in moth community composition at individual sites exceeded the variation between forest types across their three-year study, attributing this to climatic factors. This suggests that multi-year sampling does not automatically strengthen the detection of between-habitat differences, and that a single well-defined sampling period may in some cases even provide a cleaner signal of habitat effects by reducing the confounding influence of interannual climatic variability.

It is worth noting that nine species (15.5% of the total) represented unusually late records of moths that have their main flight period in summer (*Abrostola triplasia*, *Camptogramma bilineata*, *Cosmia pyralina*, *Eupithecia inturbata*, *Hoplodrina octogenaria*, *Hypsopygia costalis*, *Pandemis dumetana*, *Pleuroptya ruralis*, and *Yponomeuta plumbella*). We interpret these late records as resulting from ongoing climate change, with ever warmer periods occurring in autumn. Butterflies and moths have been a well-documented group of organisms, focusing on the effects of climate change on their behavioral, phenological and population responses [70,71,72]. Increases in temperatures and extensions of seasons have an impact on voltinism, early emergence and asynchronies [71]. A recent study in the Mediterranean region revealed that temperature and precipitation during larval development are correlated with species richness and community composition, especially for moth species with summer-developing larvae [73]. These effects of climate change will also likely affect biota in Central Europe in the future. Summer-developing larvae (first or second generation) generate adult moths that fly in the summer or autumn, causing changes in the composition of late-summer and early-autumn moth communities. Therefore, adults of species that naturally have their flying period during summertime might increasingly occur in sampling records in autumn due to phenological shifts, prolonged pupal stages, spawning of multiple generations and prolonging their flight period [70,71,73,74]. While limited in scope, our findings also seem to provide evidence of phenological responses to climate change in moth communities in the Donau-Auen National Park.

Additionally, insect sensitivity to light pollution and habitat loss from agricultural and urban changes calls for an immediate and considerate change in conservation strategies and guideline practices that focus on mitigation of light exposure and habitat connectivity, including in conservation areas [41,45,75]. Tree coverage mitigates the negative effects of artificial light at night on macro-moths by filtering light pollution, reducing its spread and intensity, and providing habitat for insects in urban areas [76]. Other practices need to be applied in suburban areas, especially those in close proximity to natural resorts and protected areas, in order to mitigate the effects of light pollution and preserve a natural sky for nocturnal diversity. Considering that outdoor illumination is a necessity, as people depend on artificial light during the night, it is more suitable to not aim for absence of artificial light but adaptation to feasible solutions. Some management options are: 1. the limitation of nightlight duration; 2. mitigation of intensity (dimming); 3. selection of the spectral composition of lights (narrow or broad); and 4. reduction in reflecting and penetrating light [22,77]. Because different target group species show varying responses to artificial light and its intensity and spectrum, corresponding measurements should be applied [30,78].

In conclusion, we demonstrated that even at a small spatial scale, the position of light traps (forest edge versus interior) had a clear effect on the abundance and richness of nocturnal insects. The forest interior harbored a significantly higher insect biomass, primarily driven by non-moth taxa. While moth community composition was strongly influenced by seasonal progression, edge sites exhibited greater community dispersion, likely due to the high number of singleton species. Surprisingly, several typically summer-flying species were detected during the autumn sampling period, suggesting a potential ongoing phenological shift associated with extended warm periods. Our findings indicate that local habitat characteristics and stressors, together with global drivers such as the climate crisis, can jointly influence insect biodiversity and abundance. Disentangling the effects of light pollution from those of habitat edge effects is methodologically challenging and complex, yet increasingly urgent for the conservation of nocturnal biodiversity, particularly in urban and peri-urban landscapes. Further research is needed to determine the relative importance of these factors and their interactions at both local and landscape scales. Future studies could extend this framework by incorporating open habitat sites alongside forest interior and edge locations, enabling a more comprehensive assessment of ecotone effects and the potential occurrence of species unique to the edge zone that are absent from both adjacent habitat types. As protected areas remain the primary tool of biodiversity conservation, it is crucial to conduct surveys both within and adjacent to their boundaries to better understand and mitigate the drivers of insect decline.

## Figures and Tables

**Table 1:** Observed moth species richness per sampling round and habitat group.

**Sampling Round**							
	1	2	3	4	5	6	7
Species Richness	41	15	12	19	13	11	13
**Trap Site**							
	Forest	Edge					
Species Richness	37	49					

**Table 2:** Species richness and Shannon diversity index for each site, aggregated over all seven replicate sampling rounds. Site Code: E = forest edge, F= forest interior.

Site Code	Species Richness	Exponential Shannon Index
E1	34	14.43
E2	25	11.7
E3	27	9.15
F1	26	10.97
F2	21	11.92
F3	26	7.96

**Table 3:** Observed richness, number of singletons, Chao1-bc species richness and estimated sampling completeness estimates by site group and season (orange > 33% singletons, green ≤ 33% singletons).

Group	Observed spp.	Singletons	Singleton %	Chao1-bc	Completeness
Overall					
Edge	49	23	46.9%	99.6	49.2%
Forest	37	11	29.7%	44.9	82.4%
Early season					
Edge	42	22	52.4%	80.5	52.2%
Forest	29	10	34.5%	35.4	81.9%
Late season					
Edge	11	3	27.3%	12.5	88%
Forest	14	2	14.3%	14.5	96.6%

**Table 4:** Significant indicator moth species (*p* < 0.005) for light trap position and season, and their indicator values as derived from the ‘indval’ function.

	Indicator Species	Indicator Value
Forest interior	*Agrochola macilenta*	0.49
	*Colotois pennaria*	0.44
	*Asteroscopus sphinx*	0.31
Late season	*Ptilophora plumigera*	0.94
	*Poecilocampa populi*	0.67
	*Operophtera brumata*	0.61
	*Asteroscopus sphinx*	0.56
	*Erannis defoliaria*	0.48
Early season	*Conistra vaccinii*	0.39
	*Agrochola litura*	0.38
	*Allophyes oxyacanthae*	0.38
	*Diloba caeruleocephala*	0.38
	*Eupithecia inturbata*	0.33
	*Agrochola nitida*	0.29
	*Pleuroptya ruralis*	0.29

## Data Availability

Data is contained within the article or Supplementary Materials.

## Supplementary Materials

The following supporting information can be downloaded at: https://www.mdpi.com/article/10.3390/insects17070670/s1, Table S1: Biomass data; Table S2: Number of individual moths per site; Table S3: Number of individual moths per site and sampling round; Table S4: Data used for iNEXT analysis; Table S5: Data used for Venn diagram; Table S6: Sites; Table S7: Data for sites and sampling rounds; File S8: R script for analysis; File S9: ReadMe file.

## Abbreviations

The following abbreviations are used in this manuscript:

NMDS	Non-metric multidimensional scaling

## Appendix A

**Table A1:** List of recorded moth species, with numbers of specimens per trap site. ‘Trophic specialization’ refers to resource use of larval stages. Polyphagous—feeding on plants in multiple families; oligophagous—larval hosts in one single plant family; monophagous—hosts in one plant genus only. Habitat affiliation: woodland—species of forest or habitats dominated by woody vegetation (shrubs, gardens, urban parks); generalist—in forested as well as open habitats; forest margins—species occurs predominantly at edges, in hedgerows or similar habitats. Information extracted from: [79,80,81,82,83,84,85,86,87].

					Forest Edge Sites			Forest Interior Sites				
Species	Family	Trophic Specialization	Hostplant Family	Habitat Affiliation	E1	E2	E3	F1	F2	F3	Collection Site Group	Sum
*Abrostola triplasia*	Noctuidae	Monophagous	Urticaceae (*Urtica*)	Generalist	0	1	0	1	0	0	both	2
*Acleris ferrugana*	Tortricidae	Oligophagous	Fagaceae (*Quercus*)	Woodland	0	0	1	0	0	0	edge	1
*Agriopis aurantiaria*	Geometridae	Polyphagous	Rosaceae, Fagaceae, Salicaceae	Woodland	0	0	0	1	4	0	forest	5
*Agriopis bajaria*	Geometridae	Polyphagous	Rosaceae, Oleaceae	Woodland	0	0	0	0	1	0	forest	1
*Sunira circellaris*	Noctuidae	Polyphagous	Salicaceae, Ulmaceae, Oleaceae	Woodland	2	3	2	2	9	10	both	28
*Agrochola laevis*	Noctuidae	Polyphagous	Fagaceae, later instars on herbs	Woodland	0	0	0	2	0	0	forest	2
*Agrochola litura*	Noctuidae	Polyphagous	Salicaceae, Fagaceae, Rosaceae, Caryophyllaceae, etc.	Generalist	1	1	1	7	3	2	both	15
*Agrochola macilenta*	Noctuidae	Polyphagous	Salicaceae, Fagaceae, Rosaceae, Ericaceae, etc.	Woodland	8	1	0	5	12	8	both	34
*Agrochola nitida*	Noctuidae	Polyphagous	Rubiaceae, Plantaginaceae, Polygonaceae	Forest margins	7	2	1	0	0	2	both	12
*Allophyes oxyacanthae*	Noctuidae	Oligophagous	Rosaceae	Woodland	1	2	2	1	1	2	both	9
*Ammoconia caecimacula*	Noctuidae	Polyphagous	Caryophyllaceae, Polygonaceae, Fabaceae, Rubiaceae, Lamiaceae, etc.	Grassland	0	1	0	0	0	0	edge	1
*Amphipyra livida*	Noctuidae	Polyphagous	Fagaceae, Asteraceae	Forest margins	0	0	0	0	1	0	forest	1
*Amphipyra pyramidea*	Noctuidae	Polyphagous	Fagaceae, Salicaceae, Ulmaceae, Rosaceae	Woodland	1	0	2	1	0	1	both	5
*Asteroscopus sphinx*	Noctuidae	Polyphagous	Fagaceae, Salicaceae, Betulaceae, Rosaceae	Woodland	2	0	1	7	5	3	both	18
*Cadra furcatella*	Pyralidae	Polyphagous	Dead dry plant matter	Synanthropic	0	0	0	0	0	1	forest	1
*Caloptilia fribergensis*	Gracillariidae	Monophagous	Sapindaceae (*Acer*)	Woodland	0	0	1	0	0	0	edge	1
*Caloptilia hemidactylella*	Gracillariidae	Monophagous	Sapindaceae (*Acer*)	Woodland	1	0	0	0	0	1	both	2
*Camptogramma bilineata*	Geometridae	Polyphagous	Rubiaceae, Rosaceae, Polygonacea	Woodland	1	0	0	0	0	0	edge	1
*Catocala electa*	Erebidae	Oligophagous	Salicaceae	Woodland (riparian)	1	0	0	0	0	0	edge	1
*Chloroclysta siterata*	Geometridae	Polyphagous	Rosaceae, Fagaceae, Salicaceae, Oleaceae, etc.	Woodland	2	0	0	2	2	2	both	8
*Cirrhia icteritia*	Noctuidae	Oligophagous	Salicaceae	Woodland	1	0	0	0	0	0	edge	1
*Cirrhia ocellaris*	Noctuidae	Oligophagous	Salicaceae (*Populus*)	Woodland (riparian)	4	0	2	1	7	0	both	14
*Colotois pennaria*	Geometridae	Polyphagous	Rosaceae, Fagaceae, Salicaceae, Ulmaceae, etc.	Woodland	4	0	5	8	11	6	both	34
*Conistra ligula*	Noctuidae	Polyphagous	Rosaceae, Fagaceae, Polygonaceae	Forest margins	1	0	0	0	0	1	both	2
*Conistra rubiginea*	Noctuidae	Polyphagous	Rosaceae, Fagaceae, Polygonaceae	Forest margins	0	0	0	0	0	1	forest	1
*Conistra vaccinii*	Noctuidae	Polyphagous	Rosaceae, Fagaceae, Betulaceae	Woodland	4	5	6	2	2	5	both	24
*Cosmia pyralina*	Noctuidae	Oligophagous	Ulmaceae, Rosaceae, Fagaceae	Woodland	0	1	1	0	0	0	edge	2
*Diachrysia chrysitis*	Noctuidae	Polyphagous	Urticaceae, Lamiaceae, Rosaceae, Boraginaceae, etc.	Generalist	0	1	0	0	0	0	edge	1
*Diloba caeruleocephala*	Noctuidae	Oligophagous	Rosaceae	Forest margins	2	1	2	3	0	3	both	11
*Dysstroma truncata*	Geometridae	Polyphagous	Rosaceae, Salicaceae, Ericaceae, Sapindaceae, etc.	Woodland	0	0	0	0	0	1	forest	1
*Epirrita dilutata*	Geometridae	Polyphagous	Fagaceae, Betulaceae, Rosaceae, Salicaceae	Woodland	26	9	10	20	27	16	both	108
*Erannis defoliaria*	Geometridae	Polyphagous	Fagaceae, Betulaceae, Rosaceae, Salicaceae	Woodland	1	1	2	12	6	12	both	34
*Eugnorisma depuncta*	Noctuidae	Polyphagous	Polygonaceae, Urticaceae, Ericaceae, Lamiaceae, Boraginaceae	Forest margins	0	1	1	0	0	0	edge	2
*Eupithecia ericeata*	Geometridae	Monophagous	Cupressaceae (*Juniperus*)	Forest margins	0	1	0	0	0	0	edge	1
*Eupithecia inturbata*	Geometridae	Monophagous	Sapindaceae (*Acer*)	Woodland	7	0	8	6	4	2	both	27
*Eupsilia transversa*	Noctuidae	Polyphagous	Fagaceae, Betulaceae, Rosaceae, Salicaceae	Woodland	0	1	0	1	0	1	both	3
*Exapate congelatella*	Tortricidae	Polyphagous	Ericaceae, Rosaceae, Fabaceae, Salicaceae, Oleaceae	Woodland	1	0	0	0	0	0	edge	1
*Griposia aprilina*	Noctuidae	Oligophagous	Fagaceae (*Quercus*)	Woodland	0	1	0	1	0	0	both	2
*Hoplodrina octogenaria*	Noctuidae	Polyphagous	Plantaginaceae, Amaranthaceae, Polygonaceae	Generalist	0	1	0	0	0	0	edge	1
*Hypsopygia costalis*	Pyralidae	Polyphagous	Dead dry plant matter	Synanthropic	1	0	0	0	0	0	edge	1
*Idaea aversata*	Geometridae	Polyphagous	Rosaceae, Rubiaceae	Generalist	1	0	0	0	0	0	edge	1
*Lithophane ornitopus*	Noctuidae	Oligophagous	Fagaceae (*Quercus*)	Woodland	0	0	0	0	1	0	forest	1
*Mniotype satura*	Noctuidae	Polyphagous	Asteraceae, Ericaceae, Scrophulariaceae, Caprifoliaceae	Woodland	2	1	1	3	0	1	both	8
*Noctua pronuba*	Noctuidae	Polyphagous	Poaceae, Asteraceae, etc.	Generalist	2	0	0	0	0	0	edge	2
*Operophtera brumata*	Geometridae	Polyphagous	Fagaceae, Rosaceae, Salicaceae, Betulaceae	Woodland	2	5	9	4	8	14	both	42
*Orgyia antiqua*	Erebidae	Polyphagous	Fagaceae, Rosaceae, Salicaceae, Betulaceae	Forest margins	0	0	0	0	1	0	forest	1
*Pandemis dumetana*	Tortricidae	Polyphagous	Urticaceae, Rosaceae, Lamiaceae, Caprifoliaceae, Asteraceae	Woodland	0	0	1	1	0	0	both	2
*Pleuroptya ruralis*	Crambidae	Polyphagous	Urticaceae, Rosaceae, Cannabaceae, Ulmaceae	Woodland	3	1	1	4	2	2	both	13
*Poecilocampa populi*	Lasiocampidae	Polyphagous	Salicaceae, Betulaceae, Fagaceae	Woodland	4	2	4	4	4	21	both	39
*Ptilophora plumigera*	Notodontidae	Monophagous	Sapindaceae (*Acer*)	Woodland	45	26	60	63	38	115	both	347
*Rhizedra lutosa*	Noctuidae	Monophagous	Poaceae (*Phragmites*)	Wetland specialist (reed beds)	0	1	0	0	0	0	edge	1
*Schrankia costaestrigalis*	Erebidae	Polyphagous	Lamiaceae, Asteraceae, Salicaceae, Solanaceae, Fabaceae,	Woodland (riparian)	1	0	0	0	0	0	edge	1
*Tiliacea aurago*	Noctuidae	Polyphagous	Sapindaceae, Fagaceae, Betulaceae, Ericaceae	Woodland	5	0	1	2	0	0	both	8
*Timandra comae*	Geometridae	Oligophagous	Polygonaceae	Generalist	1	0	0	0	0	0	edge	1
*Udea ferrugalis*	Crambidae	Polyphagous	Asteraceae, Rosaceae, Lamiacea	Generalist	2	1	1	0	0	2	both	6
*Xestia c-nigrum*	Noctuidae	Polyphagous	Urticaceae, Poaceae, Caryophyllaceae, Rosaceae, Asteraceae, etc.	Generalist	1	0	0	0	0	0	edge	1
*Xestia xanthographa*	Noctuidae	Polyphagous	Poaceae, Urticaceae, Fabaceae, Lamiaceae, Ateraceae, etc.	Generalist	0	0	1	0	0	0	edge	1
*Yponomeuta plumbella*	Yponomeutidae	Monophagous	Celastraceae (*Euonymus*)	Woodland	0	0	1	0	0	0	edge	1
Total												895
